# Plasma interleukin-6 is a potential predictive biomarker for postoperative delirium among acute type a aortic dissection patients treated with open surgical repair

**DOI:** 10.1186/s13019-021-01529-4

**Published:** 2021-05-27

**Authors:** Xiao-Chai Lv, Yong Lin, Qing-song Wu, Lei Wang, Yan-ting Hou, Yi Dong, Liang-wan Chen

**Affiliations:** 1 Department of Cardiovascular Surgery, Union Hospital, Fujian Medical University, Xinquan Road No. 29, Fuzhou, 350001 Fujian China; 2 Department of Key Laboratory of Cardio-Thoracic Surgery (Fujian Medical University), Fujian Province University, Fujian, China; 3 Department of Fujian Provincial Special Reserve Talents Laboratory, Fujian, China; 4 Department of Engineering Research Center of Tissue and Organ Regeneration, Fujian Province University, Fujian, China

**Keywords:** Aortic dissection, Postoperative delirium, Interleukin-6, Inflammatory cytokines

## Abstract

**Objectives:**

The relationship between inflammatory cytokines and postoperative delirium (POD) remains to be further investigated, especially in patients undergoing acute type A aortic dissection (AAD). Interleukin-6 (IL-6) is involved in the inflammatory process and has recently been identified as a biomarker of cerebral dysfunction. We explored the hypothesis that IL-6 was one of the critical causes of POD after surgical repair of AAD.

**Methods:**

Plasma IL-6 was measured using electrochemiluminescence technology in patients preoperatively and 24 h, 48 h, and 72 h after surgical repair of acute type A aortic dissection. After the first three postoperative days, delirium was evaluated twice daily using the Confusion Assessment Method. ROC curves were used to evaluate the ability of IL-6 measurements to distinguish POD.

**Results:**

The incidence of POD was 14.03% (31 of 221 patients). The patients in the POD group were significantly older than the patients in the non-POD group (56.48 ± 11.68 years vs 52.22 ± 10.50 years, *P* = 0.040). Plasma IL-6 concentrations were significantly higher in the POD group than in the non-POD group at three time points: preoperatively, after 24 h, and after 48 h. The AUC values corresponding to IL-6 preoperatively and 24 h after surgery were 0.73 and 0.72, respectively.

**Conclusions:**

Cerebral dysfunction after the surgical repair of AAD shows elevated stress levels and inflammatory responses. Plasma IL-6 is a potential biomarker to predict the onset of POD in acute type A aortic dissection patients following surgical repair.

## Background

Acute type A aortic dissection (AAD) usually requires urgent surgical treatment to prevent death from aortic rupture. During an aortic arch replacement operation, it is often necessary for the operation to be completed under hypothermic conditions. Moreover, aortic dissection itself may involve the blood supply of the nervous system, which may result in insufficient perfusion of the nervous system [1]. Postoperative delirium (POD) is a common complication after surgeries involving between 12 and 37% of patients undergoing aortic dissection surgery with serious effects on prognosis [2]. POD has been associated with several poor outcomes, including longer mechanical ventilation time, prolonged hospital stay, increased risk of death and greater risk of future dementia [3, 4]. It has been reported that delirium survivors are more likely to experience long-term cognitive impairment [5].

Delirium is a neuropsychiatric syndrome that is characterized by the sudden onset of altered awareness, decreased ability to maintain attention, and disorders of the mental process [6]. The pathophysiology of POD is related to postoperative neuroinflammation. Systemic inflammation in humans produces physiological and behavioral changes, one of which is characterized by cognitive decline [7]. It has been suggested that systemic inflammation may disrupt the integrity of the blood-brain barrier, resulting in endothelial dysfunction and migration of peripheral cells and their factors into the brain parenchyma, which plays a critical role in the pathophysiology of POD [8]. Hence, the levels of inflammatory markers in the circulatory system can predict the occurrence of POD to a certain extent.

It is well known that AAD surgery may induce secondary inflammatory response. Interleukin-6 (IL-6) is an important inflammatory factor. Growing evidence suggests that POD is associated with higher levels of inflammatory cytokines, such as IL-6 and C-reactive protein, in both cardiac and noncardiac surgery [8–10]. Our previous evidence has shown that IL-6 is of high predictive value for the assessment of early poor postoperative prognosis after AAD surgery [11]. Nevertheless, few studies have addressed whether POD after AAD surgery is linked to IL-6. POD is not uncommon in AAD surgery, but it is easily overlooked and often has a poor prognosis. The aim of this study was to discuss the evidence for the involvement of IL-6 in POD after AAD surgery and to highlight the relationship between POD and plasma IL-6 concentration.

## Materials and methods

The present study was approved by the Fujian Medical University Union Hospital Ethics Committee, and patients or their legal representatives provided informed consent before the operation.

### Patients

**Fig. 1:**
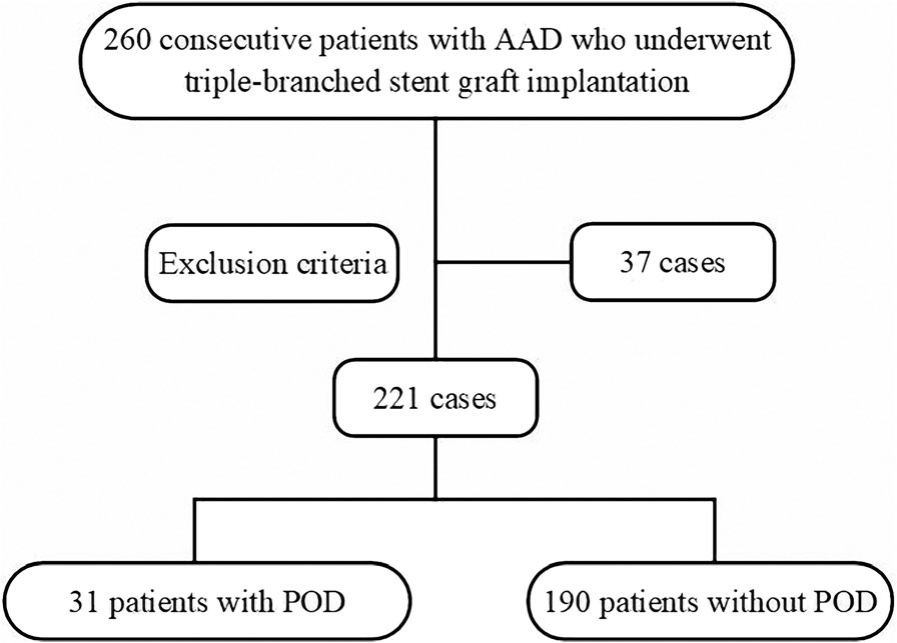
Flow chart. Two hundred and twenty-one patients with acute type A aortic dissection who underwent open surgical repair combined with triple-branched stent graft implantation were selected from 260 patients based on the exclusion criteria, and they were divided into two groups: patients with POD (*n* = 31) and patients without POD (*n* = 190). Thirty-two patients were excluded from this study according to the exclusion criteria. AAD: acute type A aortic dissection; POD: postoperative delirium

This study is a retrospective analysis of 260 consecutive patients admitted to our cardiac surgery center from March 2018 to January 2020 for open surgical repair combined with triple-branched stent graft implantation for repair of AAD. The patients were classified into two groups: patients with POD and patients without POD (Fig. 1). The inclusion criteria were as follows: (1) age greater than 18 years, (2) surgical repair combined with open triple-branched stent graft placement, and (3) signed informed consent. The exclusion criteria were: (1) prior neurological or psychiatric diseases such as dementia, stroke, schizophrenia and depression, (2) suffering from liver cirrhosis and uremia, (3) preoperative stroke or brain malperfusion,(4) patients with preoperative shock or hemodynamic instanility due to cardiac tamponade, (5) liver enzymes greater than four times the baseline, (6) hearing and/or visual impairment, (7) patients who were comatose after surgery or who died within 24 h after the surgery, (8) patients with extracorporeal membrane oxygenation therapy.

### Delirium assessment

After the first three postoperative days, delirium was evaluated twice daily in the intensive care unit (ICU) or the general ward. Delirium assessment was divided into two steps: first, the Richmond agitation-sedation score (RASS) was used to assess the level of consciousness [12]. If the RASS was lower than − 4, the score was excluded. If the patient was under sedation, the dosage of sedative drugs was adjusted, and RASS evaluation was performed again 30 min later. If the scores were above − 4, the next-stage assessment was performed, namely, the confusion assessment method for the intensive care unit (CAM-ICU) [13]. If the patient’s state of consciousness changed, it was evaluated immediately.

POD defined by CAM-ICU has the following characteristics: (1) acute onset and fluctuating course; (2) attention disorder; (3) disordered thinking; and (4) consciousness level changes. Delirium can be diagnosed by having characteristics (1), (2), (3) or (1), (2), (4). All cognitive testing was performed by two researchers who had been trained by a psychiatrist to use the CAM-ICU. Furthermore, all cognitive outcomes were validated by a third researcher who had been trained in advanced cognitive testing. During the study, the researchers who evaluated delirium were blinded to the treatment details and the serum inflammatory cytokine outcomes.

### Surgical procedure

The operation was performed under general anesthesia, and a sternal incision was made. After heparinization, cardiopulmonary bypass was established by two venous cannulas in the right atrium and two arterial cannulas in both femoral and right axillary arteries. When the nasopharyngeal temperature had decreased to 32 °C, the ascending aorta was clamped, and 4 °C cold blood cardioplegia with an elevated potassium concentration was infused directly into the left and right coronary arteries for myocardial protection.

Then, an aortic root operation was performed, and the repaired aortic root was anastomosed with a straight Dacron tube graft. After cooling to the required temperature (nasopharyngeal temperature, 20–23 °C; rectal temperature, 23–26 °C), circulatory arrest began, and bilateral selective antegrade cerebral perfusion was performed via the right axillary artery and left common carotid artery. The flow rate was approximately 8–10 mL/kg/min. The open triple-branched stent graft implantation technique has been described previously [14].

### Measurement of IL-6 concentrations

Plasma IL-6 levels of every patient were measured preoperatively and 24 h, 48 h, and 72 h after the operation using electrochemiluminescence technology (IL-6 elecscy cobas e 100, Roche, Germany). After blood was drawn, it was directly cooled and sent to our hospital laboratory for the detection of plasma IL-6.

### Data analysis and statistics

Statistical analyses were performed using SPSS (version 20.0, IBM, USA). The measurement data with normal distribution were expressed as the mean ± standard deviation, while the measurement data with nonnormal distribution were expressed as median and quartile interval. Comparisons of the two independent groups were performed with t tests or Mann-Whitney U tests (for continuous variables) and chi-square tests or Fisher’s exact tests (for categorical variables). Kruskal-Wallis tests were used to detect possible differences at different time points in each group. For each day, the preoperative day and the first three days after surgery, the discriminative ability of the single IL-6 measurement in predicting POD outcome was assessed by a receiver operating characteristic (ROC) curve and the respective area under the curve (AUC). Optimal cut-off values of IL-6 were derived from each ROC curve by identifying the point with the lowest Euclidean value. For these cut-off values, sensitivity and specificity values were calculated. A *p* value of less than 0.05 was considered statistically significant.

## Results

### Patient characteristics at baseline

Based on the inclusion and exclusion criteria, two hundred and twenty-one patients were included in this study. The excluded patients were as follows: 5 patients with prior neurological or psychiatric diseases, such as dementia, stroke, schizophrenia and depression; 3 patients with liver cirrhosis and uremia; 10 patients with preoperative stroke or brain malperfusion, and patients with preoperative shock or hemodynamic instanility due to cardiac tamponade; 8 patients with liver enzymes greater than four times the baseline; 5 patients who were comatose after the surgery or who died within 24 h after the surgery; 3 patients receiving extracorporeal membrane oxygenation therapy; and 5 patients who did not agree to participate in this clinical study.

The baseline characteristics of the patients are shown in Table 1. There were no significant differences between the two groups in terms of sex, education, body weight, height, smoking, hypertension, diabetes mellitus, coronary artery disease, ejection fraction, New York Heart Association class, or American Society of Anesthesiologists class. The patients in the POD group were significantly older than the patients in the non-POD group (56.48 ± 11.68 years vs 52.22 ± 10.50 years, *P* = 0.040), and the proportion of alcoholism in the patients in the POD group was significantly higher than that in the non-POD group (25.80% vs 6.84%, *P* = 0.003).

**Table 1:** The preoperative characteristics and blood analysis

Characteristics	All patients (***n*** = 221)	POD patients (***n*** = 31)	Non-POD patients (***n*** = 190)	***P*** values
Age (years)	52.81 ± 10.75	56.48 ± 11.68	52.22 ± 10.50	0.040*
Male (%)	162 (73.30%)	20 (64.52%)	142 (74.74%)	0.233
Education (years)	10.71 ± 2.88	10.68 ± 3.00	10.71 ± 2.87	0.945
Body weight (kg)	68.52 ± 8.28	67.48 ± 8.33	68.69 ± 8.28	0.453
Height (cm)	165.30 ± 5.39	164.97 ± 5.41	165.35 ± 5.40	0.715
Smoke (%)	80 (36.20%)	14 (45.16%)	66 (34.74%)	0.263
Alcoholism (%)	21 (9.50%)	8 (25.80%)	13 (6.84%)	0.003*
Hypertension (%)	140 (63.35%)	19 (61.29%)	121 (63.68%)	0.798
Diabetes mellitus (%)	5 (2.26%)	1 (3.23%)	4 (2.11%)	0.534
Coronary artery disease (%)	10 (4.52%)	0 (0%)	10 (5.26%)	0.364
Ejection fraction (%)	64.46 ± 5.86	66.36 ± 6.04	64.12 ± 5.80	0.126
NYHA class				0.067
I	41 (18.55%)	1 (3.23%)	40 (21.05%)	
II	139 (62.90%)	23 (74.19%)	116 (61.05%)	
III	35 (15.84%)	5 (16.13%)	30 (15.79%)	
IV	6 (2.71%)	2 (6.45%)	4 (2.11%)	
ASA class				0.314
I	0 (0.0%)	0 (0.0%)	0 (0.0%)	
II	0 (0.0%)	0 (0.0%)	0 (0.0%)	
III	0 (0.0%)	0 (0.0%)	0 (0.0%)	
IV	204 (92.31%)	30 (96.78%)	174 (91.58%)	
V	17 (7.69%)	1 (3.23%)	16 (8.42%)	
VI	0 (0.0%)	0 (0.0%)	0 (0.0%)	
Leukocytes (10^9^/l)	12.41 ± 4.43	14.65 ± 5.64	11.97 ± 4.03	0.035*
Hb (g/l)	132.94 ± 19.65	129.59 ± 29.32	133.60 ± 17.25	0.541
Hct (%)	39.79 ± 5.77	38.43 ± 8.39	39.96 ± 5.02	0.415
Creatinine (μmol/L)	96.70 ± 37.80	101.12 ± 44.29	95.89 ± 36.63	0.543
Albumin (g/l)	38.40 ± 5.09	38.46 ± 6.39	38.39 ± 4.83	0.957
Glucose (mmol/l)	5.78 ± 0.98	5.60 ± 0.89	5.81 ± 0.99	0.271
Lactate (mmol/l)	1.57 ± 0.54	1.69 ± 0.58	1.55 ± 0.53	0.175
Na + (mmol/l)	138.30 ± 3.15	138.55 ± 3.39	138.27 ± 3.12	0.654
PCT (ng/ml)	0.11 (0.05, 0.34)	0.26 (0.06, 0.01)	0.10 (0.05, 0.27)	0.082
IL-6 (pg/ml)	49.10 (30.68, 96.40)	119.20 (58.75, 170.40)	45.30 (30.00, 82.80)	0.000*
ALT (IU/L)	24.00 (15.00, 41.50)	25.00 (15.00, 49.25)	23.00 (15.00, 40.00)	0.658
AST (IU/L)	26.00 (19.00, 42.50)	29.00 (19.00, 82.25)	26.00 (19.00, 38.50)	0.284

*ASA* American Society of Anesthesiologists, *NYHA* New York Health Association, *Smoke* smoking longer than 2 months prior to surgery and more than 10 cigarettes a day, *Alcoholism* consuming 7–14 drinks per week, *Hb* hemoglobin, *Hct* hematocrit, *PCT* procalcitonin, *IL-6* interleukin-6, *ALT* glutamate aminotransferase, *AST* glutamate aminotransferase, *CRP* C-reactive protein; * *P* < 0.05

The preoperative blood analysis results are shown in Table 1. There were no significant differences in the levels of hemoglobin, hematocrit, creatinine, albumin, glucose, lactate, Na^+^, procalcitonin, glutamate aminotransferase or glutamate aminotransferase between the two groups. The level of leukocytes in the POD group was higher than that in the non-POD group (14.65 ± 5.64 vs 11.97 ± 4.03, *P* = 0.035). Compared with that in the non-POD group, the level of preoperative IL-6 in the POD group was significantly different (*P* < 0.001).

### Surgical-perioperative treatment

The perioperative and postoperative data of the two groups are presented in Table 2. There were no statistically significant differences in terms of surgery type between the two groups. No significant differences were observed for the surgical time, cardiopulmonary bypass time, aortic cross-clamp time, selective cerebral perfusion, lower-body arrest time, or lowest rectal temperature between the two groups. Although the results of our study showed that POD patients had longer ICU stays than non-POD patients (5.21 ± 2.81 vs 4.94 ± 3.03), this difference did not reach significance. The ventilation support time in the non-POD group was shorter than that in the POD group (*P* = 0.033). In addition, the length of hospital stay was significantly different between groups, with 25.23 ± 13.12 days in the POD group and 18.90 ± 8.87 days in the non-POD group (*P* = 0.014).

**Table 2:** Perioperative and postoperative data

Variables	All patients (***n*** = 221)	POD patients (***n*** = 31)	Non-POD patients (***n*** = 190)	***P*** values
Aortic root procedure				
1. Sinus repair	118 (53.39%)	14 (45.16%)	104 (54.74%)	0.322
2. Bentall procedure	47 (21.27%)	5 (16.13%)	42 (22.11%)	0.451
3. Aortic valve replacement	2 (0.90%)	1 (3.03%)	1 (0.53%)	0.261
4. David procedure	2 (0.90%)	0 (0%)	2 (1.05%)	1.00
5. CABG	9 (4.07%)	0 (0%)	9 (4.74%)	1.000
Surgery time (minutes)	303.59 ± 66.01	307.10 ± 60.13	303.00 ± 67.07	0.750
CPB time (minutes)	145.42 ± 41.14	147.58 ± 53.19	145.06 ± 38.94	0.753
Aortic cross clamp time (minutes)	48.00 ± 22.48	49.19 ± 30.68	47.80 ± 20.90	0.749
MHCA+ SCP time (minutes)	11.82 ± 4.26	12.06 ± 4.17	11.78 ± 4.28	0.731
Lowest rectal temperature (°C)	22.86 ± 0.86	22.94 ± 0.70	22.85 ± 0.88	0.600
RBC transfusion (u)	4.96 ± 1.15	5.29 ± 1.47	4.91 ± 1.09	0.175
ICU stay time (d)	4.97 ± 3.00	5.21 ± 2.81	4.94 ± 3.03	0.681
Ventilation support time (h)	55.00 (31.00, 108.50)	70.00 (41.00, 159.00)	48.50 (30.13, 99.25)	0.033*
Length of hospital stay (d)	19.81 ± 9.81	25.23 ± 13.12	18.90 ± 8.87	0.014*
DNR during hospital stay (%)	13 (5.89%)	3 (9.68%)	10 (5.26)	0.401
Mortality in hospital (%)	6 (2.70%)	2 (6.50%)	4 (2.10%)	0.199

*CABG* coronary artery bypass grafting, *CPB* cardiopulmonary bypass, *MHCA* moderate hypothermic circulatory arrest, *SCP* selective cerebral perfusion, *RBC* red blood cell, *ICU* intensive care unit, *DNR* delayed neurocognitive recovery. * *P* < 0.05

### Plasma IL-6 concentrations

**Fig. 2:**
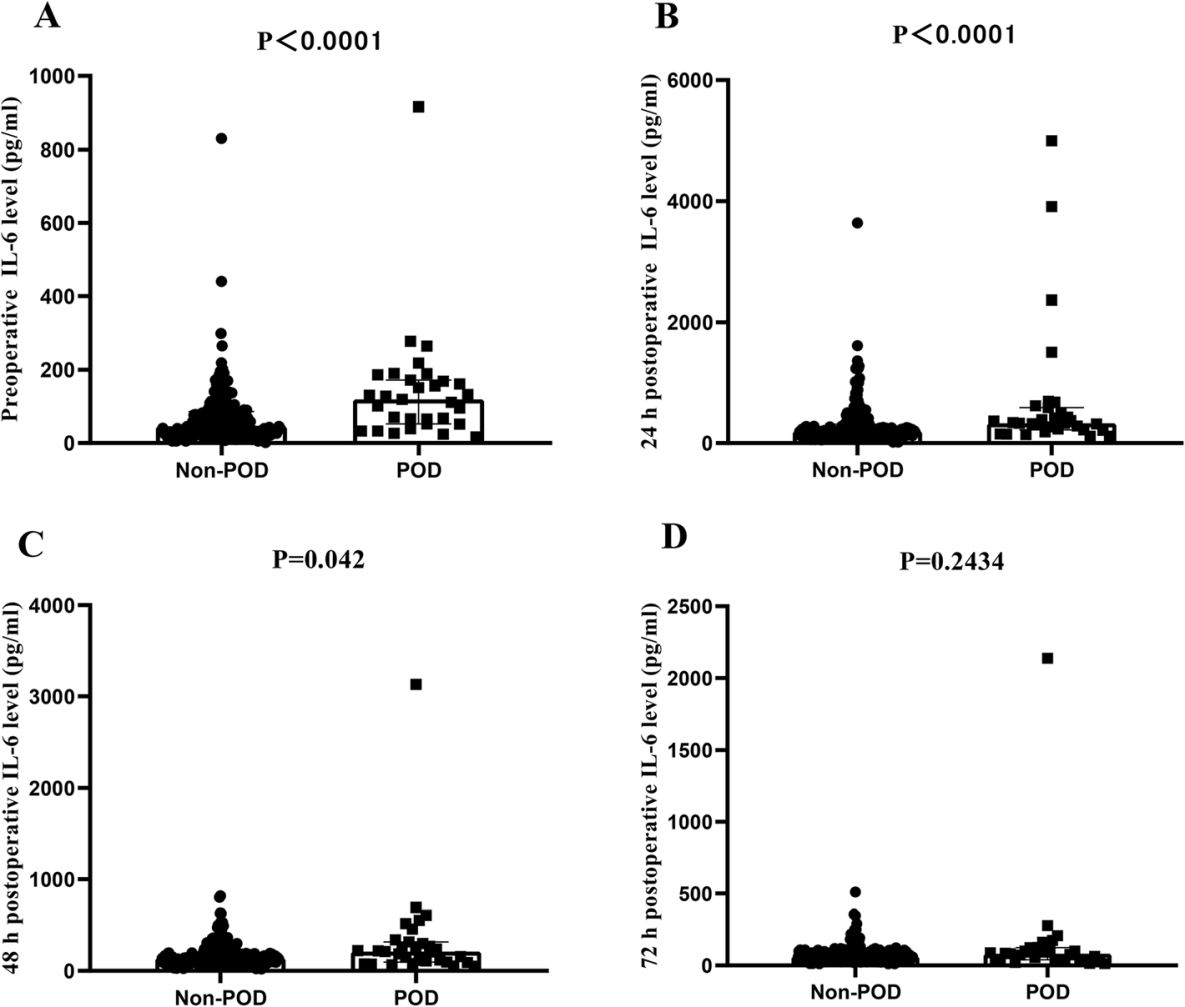
Comparison of IL-6 concentrations between the two groups at different time points. Postoperative plasma IL-6 levels were obviously higher than those preoperatively in both groups, except at 72 h after surgery. IL-6: interleukin-6

The plasma IL-6 concentrations in the patients are presented in Fig. 2. Baseline plasma concentrations of IL-6 were significantly different between the two groups (45.3 (30.0, 82.8) vs. 119.2 (58.75, 170.4) pg/ml; *P* < 0.001); compared with preoperative IL-6 concentrations, postoperative IL-6 concentrations were significantly elevated in both groups, except at 72 h after surgery. In most patients, plasma IL-6 levels increased immediately after surgical treatment, reached peak values at 24 h, and gradually decreased. Additionally, we found that plasma IL-6 concentrations were significantly higher in the POD group than in the non-POD group at two time points (329.9 (227.3, 538.5) vs 199.7 (132.1, 312.1) pg/ml after 24 h; 211.3 (103.2, 308.6) vs 141.7 (99.2, 203.1) pg/ml after 48 h; *P* < 0.05 at both time points). Moreover, the levels of IL-6 72 h after the operation tended to be higher in patients who developed delirium, yet this difference was not statistically significant.

### Plasma inflammatory cytokine measurement to predict POD

**Fig. 3:**
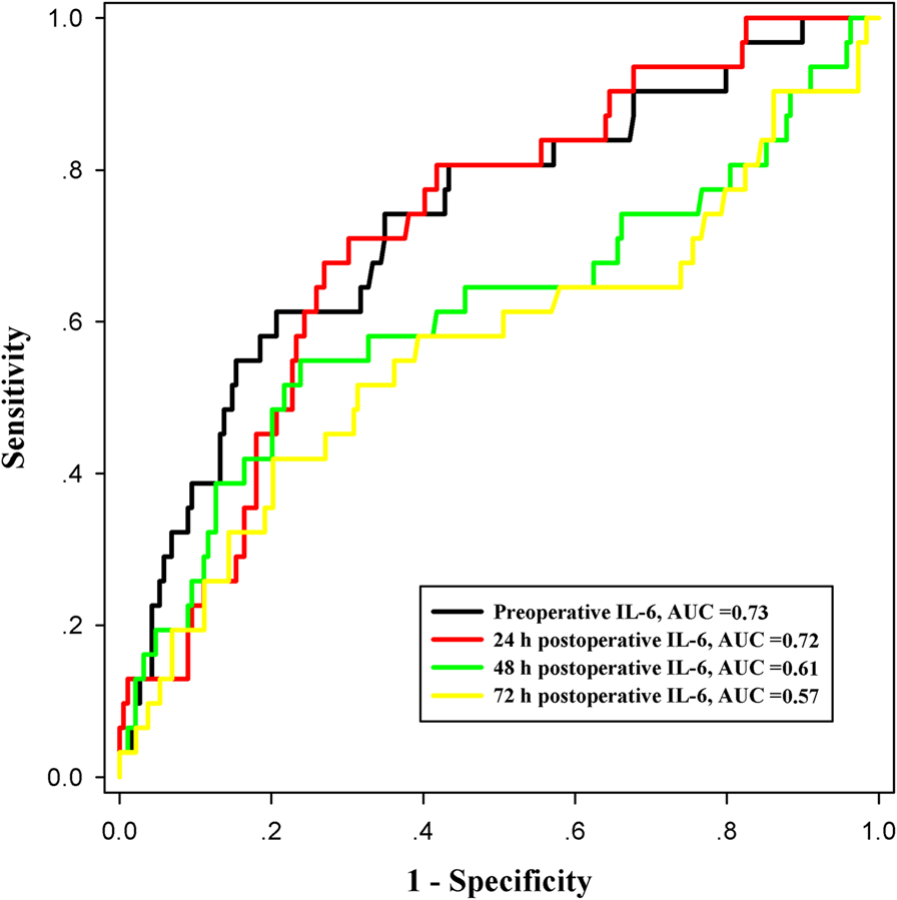
ROC curve analysis of preoperative and postoperative (24 h, 48 h and 72 h after surgery) plasma IL-6 levels for distinguishing POD. In this figure, an optimal value of plasma IL-6 levels determined preoperatively and postoperatively (24 h, 48 h and 72 h after surgery) was selected, which distinguished patients developing postoperative delirium with corresponding sensitivity and specificity values. ROC: receiver operating characteristic; POD: postoperative delirium; IL-6: interleukin-6

In Fig. 3, using the ROC curve, an optimal cutoff value of preoperative IL-6 levels (95.45 pg/ml) was chosen, which distinguished patients developing POD with 61.3% sensitivity and 79.4% specificity values. The mean AUC was 0.73 (95% CI, 0.632–0.833) for preoperative IL-6. In addition, the ROC curve selected a suitable cut-off value of 24 h postoperative IL-6 level (266.80 pg/ml) as an indicator for predicting POD, which generated a sensitivity value of 71.0% and a specificity value of 69.8%. The AUC values corresponding to IL-6 at 48 and 72 h after surgery were 0.61 and 0.57, respectively. The best thresholds at 48 and 72 h were 0.548 and 0.419 for sensitivity and 0.762 and 0.798 for specificity, respectively. In terms of the AUCs, the discriminatory ability of preoperative IL-6 levels was significantly higher than that of IL-6 levels 72 h after the operation (*P* = 0.001).

## Discussion

Previous reports have shown that POD is assumed to be the result of an inflammatory response in noncardiac surgery [15, 16]. Similar results have shown that early postoperative elevations of chemokines were associated with the development of delirium after cardiac surgery [8]. However, whether POD in aortic dissection patients after surgery is related to inflammation and the basic pathophysiological mechanism is unclear. This study show that plasma IL-6 might be associated with the inflammatory process during the development of POD in AAD patients requiring surgical repair. Moreover, plasma IL-6 could be a potential biomarker to identify AAD patients at risk for developing POD following surgical repair.

Individuals who are prone to a high degree of inflammatory response when exposed to acute stressors such as surgery or infection are at increased risk of delirium [17, 18]. It has been proposed that systemic inflammatory mediators cross the blood-brain barrier, activate the brain microglia and cause neuroinflammation [19]. Studies have shown that neuroinflammation tends to increase with aging. Wang and Shen [20] summarized the five most prominent theories to explain the development of delirium in elderly people. In the literature, age is a recognized predictor of POD occurrence [21]. Interestingly, our study also found that the patients who experienced POD were older than non-POD patients. We also found that the proportion of alcoholism was high in patients with POD. In addition, Longer operation time is one of risk factors for new-onset neurological dysfunction [22]. The finding that prolonged operative time was related to POD is not unexpected and is in accordance with our own earlier findings [23] and the observation of others [24]. Furthermore, previous evidence has suggested that temporary neurological dysfunction is a marker of long-lasting cognitive impairment [25]. The incidence of delayed neurocognitive recovery after surgery was not significantly different between the two groups at the time of hospitalization. Perhaps further associations could be found with an expanded sample size and extended follow-up time. In general, POD is a multifactorial outcome that requires early evaluation and early prevention in high-risk patients.

There are many predisposing and precipitating factors for POD [26]. Evidence has shown that patients with POD need more institutional care, have longer hospital stays, and have more care costs [23, 26]. Early evaluation of delirium onset is crucial in the ICU for early intervention, especially for AAD patients after repair surgery. These patients often experience moderate hypothermic circulatory arrest combined with selective cerebral perfusion during surgery. Moreover, perhaps they already had malperfusion of the nervous system before surgery. Thus, early detection of POD is important for these patients. Biomarkers such as peripheral inflammation cytokine, which are not affected by the use of sedatives/muscle relaxants and can be used early in the assessment process, are an important part of detection of POD.

IL-6 is a proinflammatory factor that is released when the tissue is damaged or stimulated by inflammation, and IL-6 plays local and systemic roles, producing various physiological reactions [27]. Previous studies have confirmed the role of inflammation in the development of delirium [14–16, 19]. Nevertheless, the findings of these studies are conflicting, with some studies reporting higher plasma IL-6 levels in individuals with delirium [28, 29] and others detecting different results [30]. These differences in the results may be due to the types of surgery and different methods of analyzing inflammatory markers. Our study found that plasma IL-6 values can be used to evaluate POD outcomes in AAD patients after surgery as part of a multifactorial approach. Notably, postoperative inflammatory factors were significantly increased. Moreover, the level of inflammatory factors in patients with delirium was higher than that in patients without delirium. This suggests that the release of inflammatory factors caused by surgery is a factor that induces POD.

Another purpose of this study was to test plasma IL-6 cut-off values at different time points in patients after surgery. Importantly, plasma IL-6 levels before and 24 h after surgery had higher abilities to distinguish patients at risk of POD. This finding indicates that the diagnostic value of IL-6 levels at these two time points is reliable. The AUC values of IL-6 levels at 48 h and 72 h after surgery were greater than 0.5 but less than 0.7, indicating that although the diagnostic value of IL-6 levels at these two time points were both significantly higher than 0.5, the diagnostic value was still relatively low. The reason why the predictive ability of IL-6 gradually decreased 48 and 72 h after the operation may be related to the gradual recovery of cognitive function in some patients.

There are some limitations to our study. First, the retrospective design of our study and the lack of randomization may lead to possible selection bias. Second, our study was limited to examination of a single marker of inflammation, IL-6. Moreover, it will be important in future studies to examine the associations of IL-6 with delirium duration and feature severity. Third, the sample size in patients with POD is small, which should be considered when interpreting the results.

## Conclusion

We found that in AAD patients undergoing surgery, plasma IL-6 measured at several timepoints was associated with delirium. Moreover, plasma IL-6 could be a potential biomarker to predict the onset of POD in AAD patients following surgical repair.

## Data Availability

The datasets generated and/or analysed during the current study are not publicly available due to the confidentiality agreement of our institution but are available from the corresponding author on reasonable request.

## Abbreviations

AAD: Acute type A aortic dissection
POD: Postoperative delirium
IL-6: Interleukin-6
ICU: Intensive care unit
RASS: Richmond agitation-sedation score
CAM-ICU: confusion assessment method for the intensive care unit
ROC: receiver operating characteristic
AUC: area under the curve
